# Spontaneous Gait Transitions of Sprawling Quadruped Locomotion by Sensory-Driven Body–Limb Coordination Mechanisms

**DOI:** 10.3389/fnbot.2021.645731

**Published:** 2021-07-30

**Authors:** Shura Suzuki, Takeshi Kano, Auke J. Ijspeert, Akio Ishiguro

**Affiliations:** ^1^Research Institute of Electrical Communication, Tohoku University, Sendai, Japan; ^2^Japan Society for the Promotion of Science, Tokyo, Japan; ^3^Biorobotics Laboratory, École Polytechnique Fédérale de Lausanne, Lausanne, Switzerland

**Keywords:** salamander locomotion, body-limb coordination, gait transition, decentralized control, sensory feedback control

## Abstract

Deciphering how quadrupeds coordinate their legs and other body parts, such as the trunk, head, and tail (i.e., body–limb coordination), can provide informative insights to improve legged robot mobility. In this study, we focused on sprawling locomotion of the salamander and aimed to understand the body–limb coordination mechanisms through mathematical modeling and simulations. The salamander is an amphibian that moves on the ground by coordinating the four legs with lateral body bending. It uses standing and traveling waves of lateral bending that depend on the velocity and stepping gait. However, the body–limb coordination mechanisms responsible for this flexible gait transition remain elusive. This paper presents a central-pattern-generator-based model to reproduce spontaneous gait transitions, including changes in bending patterns. The proposed model implements four feedback rules (feedback from limb-to-limb, limb-to-body, body-to-limb, and body-to-body) without assuming any inter-oscillator coupling. The interplay of the feedback rules establishes a self-organized body–limb coordination that enables the reproduction of the speed-dependent gait transitions of salamanders, as well as various gait patterns observed in sprawling quadruped animals. This suggests that sensory feedback plays an essential role in flexible body–limb coordination during sprawling quadruped locomotion.

## 1. Introduction

Quadruped animals exhibit a high agility and adaptability to terrestrial environments. These locomotor abilities are achieved by coordinating their legs and other body parts, such as the trunk, head, and tail (i.e., through body–limb coordination). For instance, the bending of a cheetah's body improves its speed (Hildebrand, 1959), a horse's nodding reduces metabolic costs (Loscher et al., 2016), and the undulation of a salamander's tail facilitates dynamic balance (Bicanski et al., 2013b). These examples suggest that the body–limb coordination mechanisms play an essential role in animal locomotor skills. Decoding the body–limb coordination mechanisms will contribute to the design of highly functional legged robots and help understand the motor control of legged animals.

The salamander is an amphibian and is well-suited for investigating the body–limb coordination mechanisms because it exhibits a flexible body–limb coordination dependent on locomotion speed (Ashley-Ross, 1994). At slow speeds, salamanders show lateral-sequence walking gait (L-S walk) with standing waves of lateral body undulation in which the body oscillates synchronously while some points act as “nodes” and do not move. At higher speeds, they exhibit a walking trot gait with first standing waves (at medium speeds) and then traveling waves (at high speeds) of lateral undulation in which all body parts oscillate laterally, propagating the waves rostrocaudally. Despite this flexible body–limb coordination, the locomotor nervous systems of salamanders are simpler than those of mammals in that they have fewer neurons and less differentiated structures (Chevallier et al., 2008; Bicanski et al., 2013b). Therefore, salamanders likely possess flexible and simple body–limb coordination mechanisms.

The locomotion of salamanders and other vertebrate animals is controlled by distributed neural networks, called central pattern generators (CPGs), and sensory feedback from peripheral nerves (Cabelguen et al., 2003; Ryczko et al., 2020). In particular, decerebrate salamander experiments showed that neural communication between CPGs is responsible for coordinating axial and limb movements. Base on these finding, CPG networks have been modeled, and salamander locomotion investigated through numerical simulations and robot experiments (Ijspeert, 2001, 2020; Ijspeert et al., 2007; Harischandra et al., 2010, 2011; Bicanski et al., 2013a; Crespi et al., 2013; Liu et al., 2018). Most previous studies used an oscillator model with inter-oscillator couplings in which oscillators represent CPGs and inter-oscillator couplings represent neural communications between CPGs. These studies designed inter-oscillator couplings to coordinate axial and limb movements and reproduced various behaviors, such as walking, swimming, and turning (Ijspeert, 2001; Ijspeert et al., 2007; Bicanski et al., 2013a; Crespi et al., 2013; Liu et al., 2018). These studies intensively investigated the body–limb coordination mechanisms based on inter-oscillator couplings, and less attention was paid to the role of sensory feedback in body–limb coordination. Harischandra et al. (2011) proposed a CPG model with stretch sensory feedback and showed that sensory feedback contributes to gait generation and transition. However, the main focus of this study is interlimb coordination, as opposed to body–limb coordination. The role of sensory feedback in body–limb coordination remains elusive.

We aim to understand the contribution of sensory feedback to body–limb coordination. We previously proposed a decentralized control model with cross-coupled sensory feedback from the body to limb, and vice versa, in simulated and real sprawling quadruped robots (Suzuki et al., 2019, 2021). Body–limb coordination was successfully established by sensory couplings without inter-oscillator couplings. These studies also suggested that sensory feedback provides rapid convergence to a stable gait, easy parameter tuning, and high robustness against leg failure and morphological changes. However, the results cannot explain the body–limb coordination mechanisms responsible for gait transition because of the simplified body structure in which the body trunk had only one degree of freedom.

In this study, we investigate the mechanisms for coordination between the legs and a flexible elongated trunk and aim to reproduce the speed-dependent gait transition of salamanders. To this end, we extend our previous model to simulate a salamander robot with a multi-segmented trunk. The simulation results show that the proposed model can reproduce the gait transition between a standing wave pattern at low speed and a traveling wave pattern at high speed, by changing only one parameter related to the command from the brain. The model also reproduces several gait patterns observed in other sprawling quadruped animals by changing the sensory feedback strength. These results suggest that, in addition to inter-oscillator couplings (which are known to exist in the salamander spinal cord), sensory feedback could play an essential role in flexible body–limb coordination underlying sprawling quadruped locomotion.

The remainder of this paper is structured as follows. Section 2 contains a description of a decentralized control for body–limb coordination and details the effects of sensory feedback rules. Section 3 contains an outline of the simulation experiments and the results. In section 4, the potential role of sensory feedback in body–limb coordination is discussed, and recommendations for future studies are presented.

## 2. Model

### 2.1. Body

**Figure 1:**
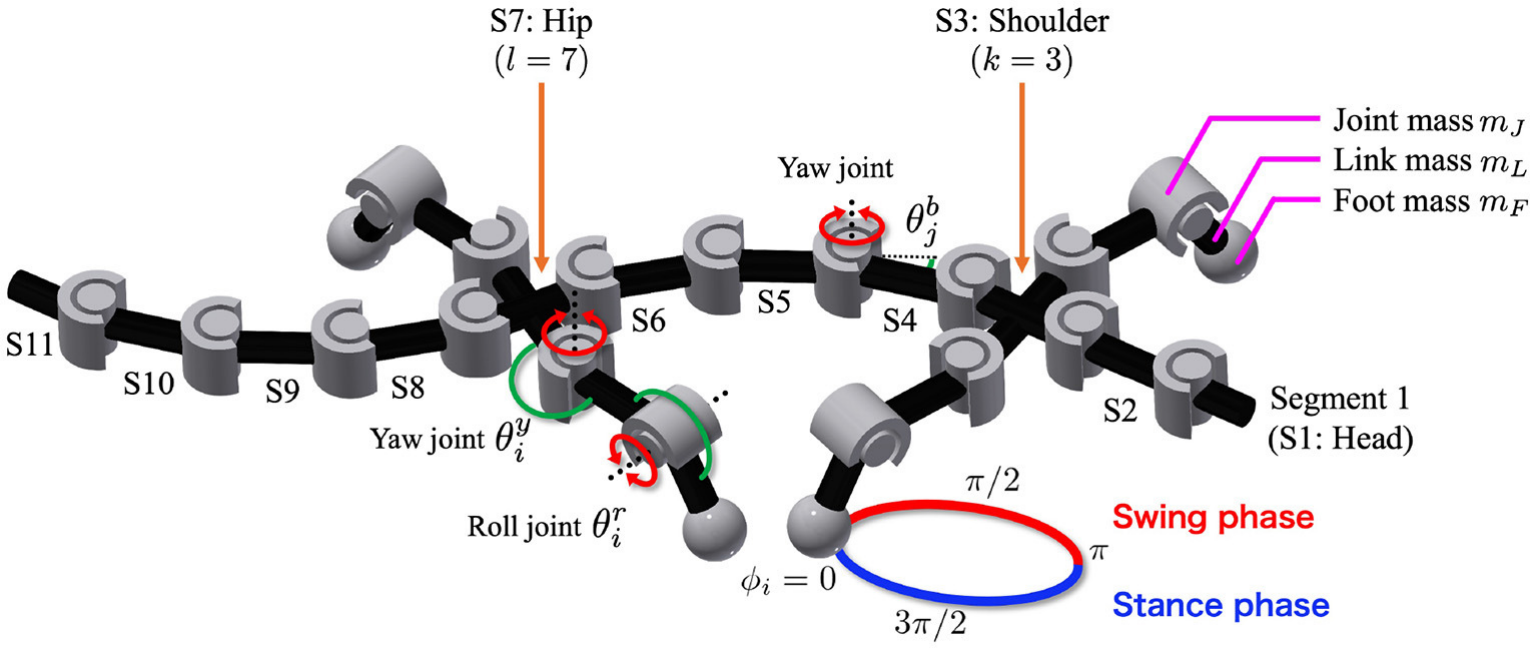
Body model. The trunk has *n* − 1 actuated degrees of freedom (DoFs), and $\theta_{j}^{b}$ denotes the angle of the *j*-th DoF from the head. The fore- and hind-legs are attached on both sides of the *k*-th and *l*-th segments, respectively (in the figure, *n* = 11, *k* = 3, and *l* = 7). Each leg has two DoFs controlled by phase oscillators. The subscript *i* denotes the leg identifier: (1, left fore; 2, right fore; 3, left hind; and 4, right hind), and $\theta_{i}^{y}$ and $\theta_{i}^{r}$ are the angles of the leg joints in the yaw and roll directions, respectively. The circle around the right foreleg shows the leg trajectory based on the oscillator phase ϕ_*i*_. Variables *m*_*J*_, *m*_*L*_, and *m*_*F*_ are the masses of each joint, link, and foot, respectively.

The body consists of *n* trunk segments and four legs, as shown in Figure 1. The segments are concatenated via yaw hinge joints with a parallel combination of a rotary actuator, passive spring, and passive damper. The fore- and hind-legs are attached on both sides of the *k*-th and *l*-th segments, respectively. Each leg has two rotary actuators in the yaw and roll directions, controlled by phase oscillators.

Each foot tip has a force sensor that detects the normal force from the ground, and each trunk joint has angle and torque sensors. The angle sensors detect the angle $\theta_{j}^{b}$ of the *j*-th trunk joint from the head. Here, the *j*-th trunk joint connects the *j*-th and *j* + 1-th segments from the head. The variable $\theta_{j}^{b}$ is positive when the trunk joint bends to the right, as shown in Figure 1. The torque sensors detect the torque generated by the rotary actuators at the trunk joint.

### 2.2. Control Algorithm

**Figure 2:**
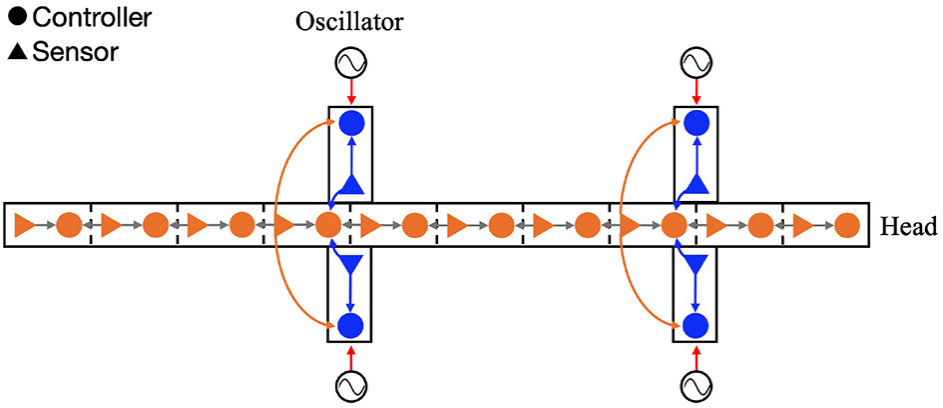
Configuration of the feedback network. The circles and triangles represent the controllers and sensors, respectively. Each leg controller has one phase oscillator. The arrows show the four types of sensory feedback; blue indicate the force feedback from limb to limb and limb to body, orange indicates the torque feedback from body to limb, and gray indicates the angle feedback from body to body.

The proposed decentralized control algorithm is an extension of our previous study (Suzuki et al., 2019). The controller is composed of oscillators, which represent CPGs. In order to focus on the potential role of sensory feedback as synchronization mechanism, inter-oscillator couplings are not modeled here; instead, nearby body parts are coupled through sensory feedback (Figure 2). The sensory feedback consists of the following four feedback rules:

1. Force feedback from limb to limb
2. Torque feedback from body to limb
3. Force feedback from limb to body
4. Angle feedback from body to body

The first rule is responsible for coordinating the four legs as they move forward while supporting the body. The second and third rules comprise the cross-coupled feedback that establishes self-organized body–limb coordination. The fourth rule coordinates the lateral undulations of the multi-segmented body trunk. Through the interplay of these rules, it is expected that the model will generate flexible locomotion patterns. The following section describes each sensory feedback control in detail.

#### 2.2.1. Leg Control

A phase oscillator is implemented in each leg, and its phase determines the target angle of the rotary actuators in the yaw and roll directions as follows:

(1) $$\begin{array}{l} {\bar{\theta }}_{i}^{y}=C_{0}^{y}-C_{amp}^{y}\cos\phi_{i},\\ {\bar{\theta }}_{i}^{r}=C_{0}^{r}-C_{amp}^{r}\sin\phi_{i}, \end{array}$$

where ${\bar{\theta }}_{i}^{y}$ and ${\bar{\theta }}_{i}^{r}$ denote the target angles, $C_{0}^{y}$ and $C_{0}^{r}$ represent the neutral angles, $C_{amp}^{y}$ and $C_{amp}^{r}$ represent the amplitudes of the yaw and roll actuators, respectively (Figure 1); ϕ_*i*_ is the oscillator phase and the subscript *i* denotes the leg identifier (1: left fore, 2: right fore, 3: left hind, and 4: right hind). When 0 < ϕ_*i*_ < π, the leg tends to be in the swing phase; otherwise, it tends to be in the stance phase. The time evolution of the phase is described as follows:

(2) $${\dot{\phi }}_{i}=\omega +f_{LL,i}+f_{BL,i},$$

(3) $$f_{LL,i}=-\sigma_{LL}\tanh(\rho_{LL}N_{i})\cos\phi_{i},$$

(4) $$f_{BL,i}=\left\{ \begin{array}{l} +\sigma_{BL}\tanh(\rho_{BL}\tau_{k}^{b})\cos\phi_{i}\;(i=1)\\ -\sigma_{BL}\tanh(\rho_{BL}\tau_{k}^{b})\cos\phi_{i}\;(i=2)\\ +\sigma_{BL}\tanh(\rho_{BL}\tau_{l}^{b})\cos\phi_{i}\;(i=3)\\ -\sigma_{BL}\tanh(\rho_{BL}\tau_{l}^{b})\cos\phi_{i}\;(i=4), \end{array}\right.$$

where ω [rad/s] denotes the intrinsic angular velocity of the phase oscillators; and σ_*LL*_ [rad/s], ρ_*LL*_ [1/N], σ_*BL*_ [rad/s], and ρ_*BL*_ [1/(N·m)] are the weights of the sensory feedback terms; and *N*_*i*_ [N] represents the normal force detected at the foot tip. Further, $\tau_{k}^{b}$ and $\tau_{l}^{b}$ [N·m] represent the torque generated by the *k*-th and *l*-th trunk actuators, respectively.

Equation (3) relates to the limb-to-limb feedback. The local feedback rule was proposed by Owaki et al. (2013). It generates adaptive interlimb coordination in response to the speed and physical properties of the robot (Owaki et al., 2013; Owaki and Ishiguro, 2017). Based on the sensory feedback effect, the oscillator phase is modulated to 3π/2 when *N*_*i*_ > 0. When the leg supports the body, the foot obtains a higher ground reaction force, that is, a higher *N*_*i*_. Thus, this feedback implies that the leg remains on the ground when it supports the body. The local sensory information, denoted by *N*_*i*_, describes the extent to which a specific leg provides support to the body, and it also indicates how much other legs are currently contributing to supporting the body. Using such sensory information, this feedback can generate adaptive interlimb coordination without neural communication between the legs.

**Figure 3:**
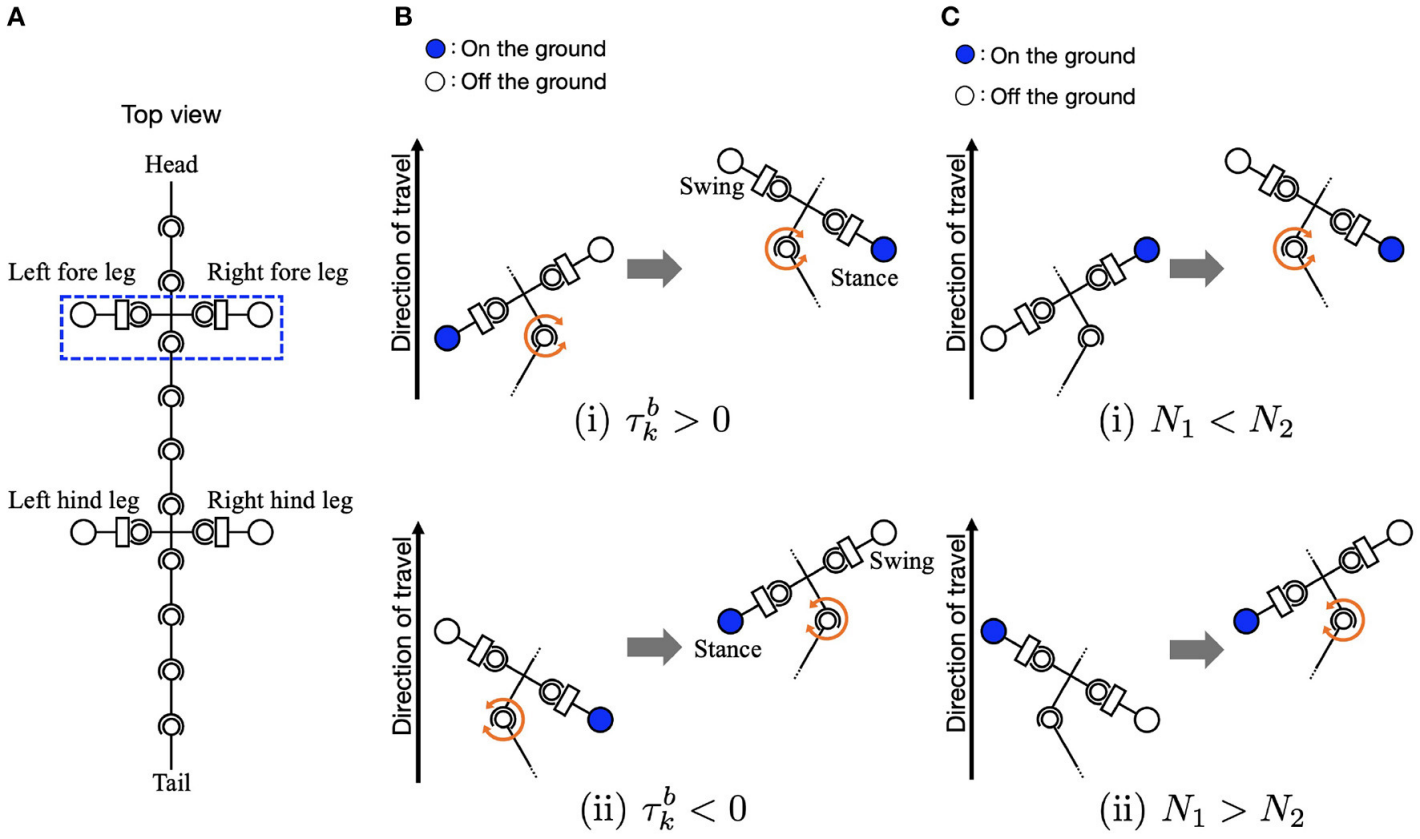
Schematic of body-limb sensory feedback. **(A)** Schematic of the salamander robot model from the top view. The squared region around the forelegs indicates the body part illustrated by **(B,C)** to explain the feedback effect. **(B)** Body-to-limb sensory feedback mechanism: (i) the *k*-th trunk actuator bends the body to the right ($\tau_{k}^{b}>0$) and (ii) the *k*-th trunk actuator bends the body to the left ($\tau_{k}^{b}<0$). When the *k*-th trunk actuator bends the body to the right, the left foreleg oscillator phase is modulated toward π/2 (to swing), and the right foreleg oscillator phase is modulated toward 3π/2 (to stance), and vice versa. **(C)** Limb-to-body sensory feedback mechanism: (i) the right foreleg is on the ground (*N*_2_ > 0) and (ii) the left foreleg is on the ground (*N*_1_ > 0).

Equation (4) relates to the body-to-limb feedback (Figures 3A, B). When the *k*-th trunk actuator bends the body to the right ($\tau_{k}^{b}>0$), the oscillator phase of the left foreleg is modulated to π/2 to lift the legs, and the oscillator phase of the right foreleg is modulated to 3π/2 to place the legs on the ground. By phase modification, the left foreleg lifts from the ground, and the right foreleg is anchored to the ground. This facilitates the *k*-th trunk actuator to bend the body to the right ($\theta_{k}^{b}>0$), and the robot moves forward when the anchored legs serve as a pivot. Similarly, the oscillator phases of the hind legs are modulated by the torque of the *l*-th trunk actuator.

#### 2.2.2. Body Control

The torques at the trunk actuators are described as follows:

(5) $$\tau_{j}^{b}=f_{LB,j}+f_{BB,j},$$

(6) $$f_{LB,j}=\{\begin{array}{ll} \sigma_{LB}\tanh\{\rho_{LB}(N_{2}-N_{1})\}\; & \left(j=k\right)\\ \sigma_{LB}\tanh\{\rho_{LB}(N_{4}-N_{3})\}\; & \left(j=l\right)\\ 0\; & \text{otherwise}, \end{array}$$

(7) $$f_{BB,j}=-\sigma_{BB}\tanh\{\rho_{BB}(\theta_{j}^{b}-\theta_{j-1}^{b})\},$$

where $\theta_{j}^{b}$ is the actual angle of the trunk actuator. The variables σ_*LB*_ [N·m] and ρ_*LB*_ [1/N], σ_*BB*_ [N·m], and ρ_*BB*_ [1/rad] represent the weights of the sensory feedback.

Equation (6) relates to the limb-to-body feedback. The sensory feedback effect is such that the *k*-th and *l*-th trunk segments bend in response to ground contacts, as shown in Figures 3A, C. When the left foreleg is on the ground (*N*_1_ > 0), the *k*-th actuator bends the body to the left ($\tau_{k}^{b}<0$). Similarly, when the right foreleg is on the ground (*N*_2_ > 0), the *k*-th trunk actuator bends the body to the right ($\tau_{k}^{b}>0$). The interactions of the sensory feedback from body to limb and limb to body establish the relationship between the legs and trunk, providing longer strides and more powerful pushing-off against the ground. The interactions of the body-to-limb and limb-to-body feedback establish the relationship between the legs and trunk, providing longer strides and more powerful pushing-off against the ground (Suzuki et al., 2019, 2021).

Equation (7) relates to the body-to-body feedback. The local feedback rule is based on the curvature derivative control proposed in a previous study for snake-like locomotion (Date and Takita, 2007). It generates a torque proportional to the curvature derivative of the body curve such that the lateral body undulation propagates posteriorly. As reported in a previous study (Kano and Ishiguro, 2020), the control with additional sensory feedback can generate versatile undulation patterns. Therefore, it can potentially generate flexible, sensory-driven, intersegmental coordination, as an alternative to inter-oscillator couplings (as modeled in Ijspeert et al. (2007) for instance). The interplay between limb-to-body and body-to-body feedback arranges the waveform of the lateral body undulations.

## 3. Simulation Results

We conducted simulation experiments using the Open Dynamics Engine, which is an open-source library for simulating rigid body dynamics (Smith, 2005). Each trial was conducted on flat terrain for 60 s, with the oscillator phases initially set to random. The body size and weight were determined by considering those of a salamander robot developed as a prototype in our previous study (Suzuki et al. 2019b). The angular frequency and amplitude of the legs were chosen with physically plausible values. The other parameters were determined by trial and error, referring to the parameter sets of our previous simulation study [Suzuki et al., 2019]. The simulation time step was set to 0.01 s, and the control commands were updated at each time step. The results are provided in the following sections. We first show that the speed-dependent gait transition of salamanders can be successfully reproduced (section 3.1). Next, we demonstrate that two other gait patterns observed in other sprawling quadruped animals can be reproduced (section 3.2). Finally, we clarify which parameters affect the exhibited gait pattern by changing the feedback strength (section 3.3) and body parameters (section 3.4).

### 3.1. Speed-Dependent Gait Transition of Salamanders

**Figure 4:**
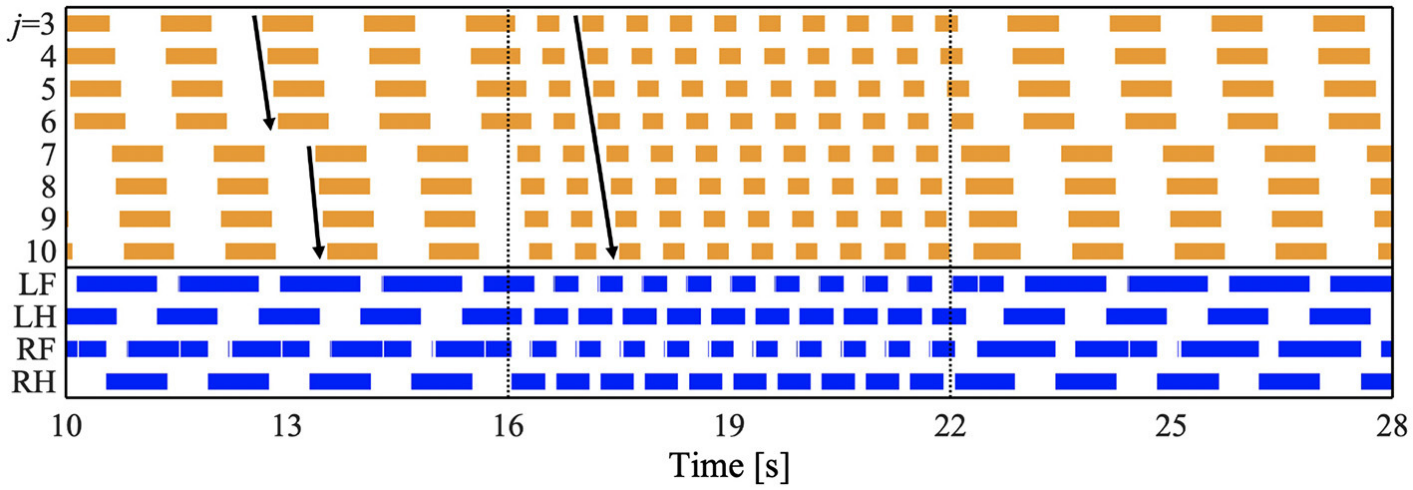
Spontaneous gait transition from L-S walk with standing waves to walking trot with traveling wave and vice versa. The upper graph represents the lateral flexion of the trunk joint, wherein the colored region denotes the period when the trunk joint bends to the right ($\theta_{j}^{b}>0$). The lower graph represents the gait diagram, wherein the colored region denotes the period when the foot is in contact with the ground (*N*_*i*_ > 0). We set the parameter ω from 1.8 π to 3.8 π [rad/s] at period 16 [s], and from 3.8 π to 1.8 π [rad/s] at period 22 [s]. We confirmed that the gait transition was observed for any initial oscillator phase (for all 10 trials).

To investigate whether the proposed model can reproduce the speed-dependent gait transition of salamanders, we performed a simulation by changing the parameter ω from 1.8 π to 3.8 π [rad/s] at period 16 [s] and from 3.8 π to 1.8 π [rad/s] at period 22 [s]. Figure 4 and Supplementary Video 1 show the results. The upper graph represents the lateral flexion of the trunk joint, wherein the colored region denotes the period when the trunk joint bends to the right ($\theta_{j}^{b}>0$). The lower graph represents the gait diagram, wherein the colored region denotes the period when the foot is in contact with the ground (*N*_*i*_ > 0).

For ω = 1.8π, the bending of the body trunk (*j* = 3−6) is antiphase to that of the tail (*j* = 7−10), as shown in Figure 4. This pattern is a standing wave with nodes at the shoulder and the hip, similar to that of a salamander walking (Ashley-Ross, 1994). Next, the feet touched down in the following order: right hind (RH), right fore (RF), left hind (LH), and left fore (LF). The mean and standard deviation (SD) of the duty factor were 69.3 and 0.55%, respectively. The mean and SD of the diagonality were 21.9 and 1.26%, respectively. These values were calculated within 10–16 [s] for each of the 10 trials. The duty factor is the time percentage at which one foot spends in the stance phase during a gait cycle, and diagonality is the percentage of the cycle period by which the left/right hind footfall precedes the left/right fore-footfall. Thus, the gait was classified as a lateral-sequence (L-S) walk, according to Hildebrand's gait classification (Hildebrand, 1965; Cartmill et al., 2002). This gait was observed in salamander's slow-speed walking (Ashley-Ross, 1994). In conclusion, for ω = 1.8π, the model reproduced the bending and footfall patterns of a salamander's slow-speed walking.

For ω = 3.8π, the flexion duration moved posteriorly and continuously (Figure 4), indicating a traveling wave. The footfall pattern is such that the diagonally opposite feet were nearly synchronized. The mean and SD of the duty factor were 64.5% and 9.74 × 10^−2^, respectively. The mean and SD of the diagonality were 48.0 and 1.97%, respectively. These values are calculated within 18–22 [s] for each of the 10 trials. Thus, the gait was classified as a walking trot according to Hildebrand's gait classification (Hildebrand, 1965; Cartmill et al., 2002). The bending and footfall patterns were observed in a salamander's high-speed walking (Ashley-Ross, 1994). Therefore, the model also reproduced the gait pattern of a salamander's high-speed walking for ω = 3.8π.

When changing ω from 1.8 π to 3.8 π at period 16 [s], the gait pattern spontaneously and smoothly changed from a L-S walk with standing waves to a walking trot with traveling waves, as shown in Figure 4. Similarly, the reverse gait transition (from walking trot to L-S walk) was observed when changing ω from 3.8 π to 1.8 π at period 22 [s]. We confirmed that the gait transition was observed for any initial oscillator phase (for all 10 trials). Thus, the proposed model successfully reproduced the speed-dependent gait transition of salamanders, by simply changing the ω parameter.

**Figure 5:**
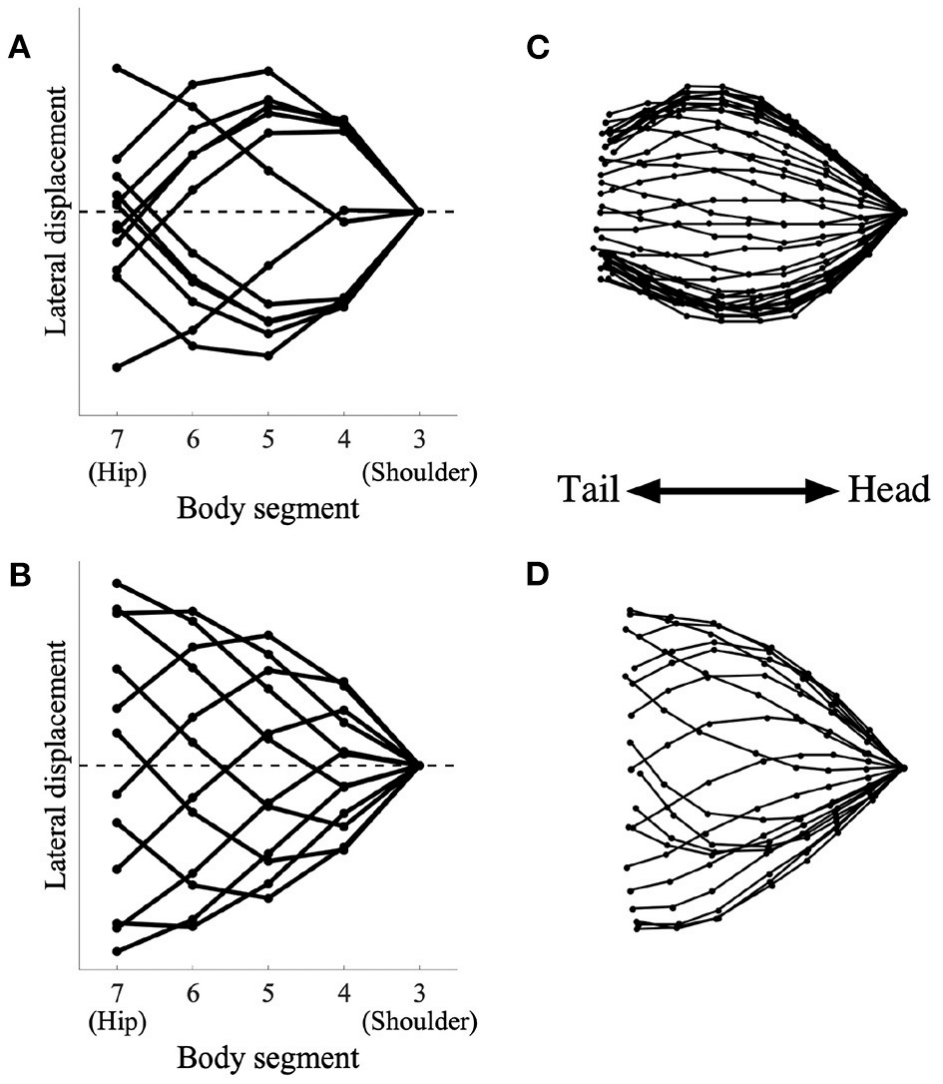
Simulated robot (left) and salamander *D. teneborosus* (right) during locomotion. The stick figures were made by connecting the positions of the body parts over the midline. All stick figures throughout one gait cycle were superimposed by lining them up on the anteriormost part of the body trunk. **(A)** Simulated robot for ω = 1.8π, **(B)** simulated robot for ω = 3.8π, **(C)** *D. teneborosus* while walking, **(D)** *D. teneborosus* while trotting. **(C,D)** Adapted from the Ashley-Ross's study (Ashley-Ross, 1994), with permission.

We then analyzed the lateral bending waveform of each gait pattern and compared it with that of salamanders. Figure 5 shows the comparison of the body waveform between the simulated robot and the salamander, *Dicamptodon teneborosus* (Ashley-Ross, 1994). In Figures 5A,B, the stick figures were made by connecting the lateral positions of the body segments from the shoulder (*j* = 3) to the hip (*j* = 7) in the simulated robot for ω = 1.8π (Figure 5A) and ω = 3.8π (Figure 5B), respectively. All stick figures throughout one gait cycle were superimposed by lining them up on the shoulder segment. This analysis refers to Ashley-Ross's study (Ashley-Ross, 1994), and Figures 5C,D were adapted from the analysis of a salamander walking and trotting conducted in this study. These stick figures were made by connecting the marker point over the midline from the pectoral girdle to the pelvic girdle while walking (Figure 5C) and trotting (Figure 5D). All stick figures throughout one gait cycle were superimposed by lining them up on the anteriormost midline marker dot.

Figure 5A shows the body waveform alternate between two stable curve configurations; the curve features a half-wavelength from the shoulder and hip. This pattern is a standing wave with nodes at the shoulder and the hip, and is similar to that of a salamander's walking, as shown in Figure 5C. Figure 5B shows that the body waveform has no nodes; the trunk does not follow a simple side-to-side bending pattern (such as in Figure 5A). This pattern is a traveling wave, and is also similar to that of a salamander's trotting, as shown in Figure 5D. These results suggest that the model certainly generates two types of body waveforms, namely, standing and traveling waves; these waveforms are qualitatively similar to those exhibited by a salamander.

### 3.2. Reproduction of Gait Patterns Observed in Other Species

While salamanders exhibit a L-S walk with standing waves of lateral body undulation and walking trot gait with standing or traveling waves of, different gait patterns have been observed in other species that exhibit sprawling locomotion (Ritter, 1992). In this subsection, we demonstrate that the proposed model can reproduce such patterns by changing the control parameters.

#### L-S Walk With Intermediate Waves

**Figure 6:**
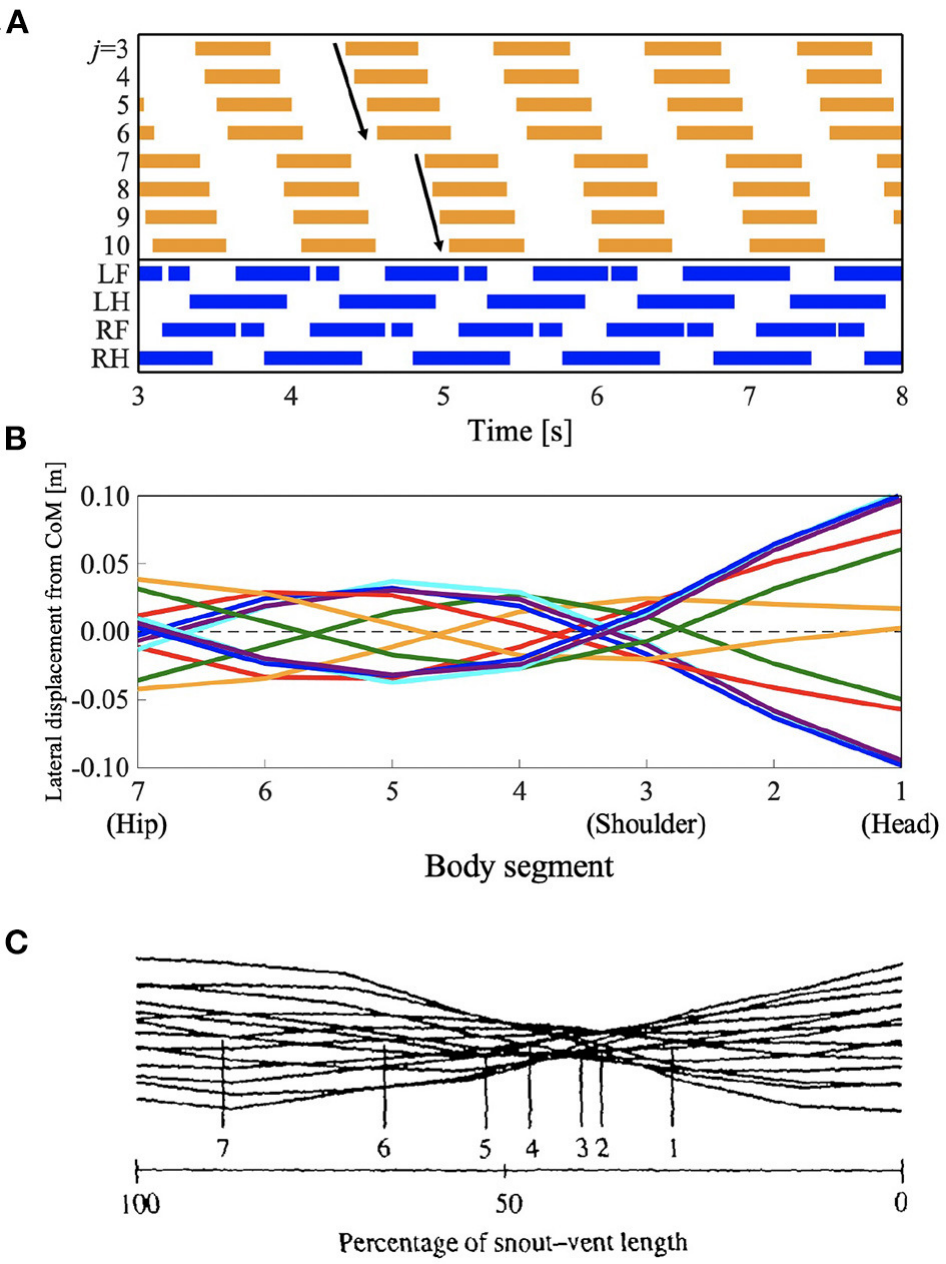
Lateral-sequence walking with intermediate waves of lateral bending. The proposed model without any modified parameter except for the control parameter ω reproduced the gait pattern, as listed in Table 1. **(A)** The upper graph represents the lateral flexion of the trunk joint, wherein the colored region denotes the period when the trunk joint bends to the right ($\theta_{j}^{b}>0$). The lower graph represents the gait diagram, wherein the colored region denotes the period when the foot is in contact with the ground (*N*_*i*_ > 0). **(B)** Waveform of lateral bending of the simulated robot. The superimposed figure was made from 12 stick figures. The stick figures were produced by connecting the lateral displacement of body segments from the center of mass (CoM). The moment of each stick figure is time-shifted every 1/12 gait cycle, and the line colors show the order of the stick figures (red: 1st and 7th; orange: 2nd and 8th; green: 3rd and 9th; cyan: 4th and 10th; blue: 5th and 11th; and violet: 6th and 12th). Note that the 1st and 7th stick figures are time-shifted by a half period of the gait cycle, thereby being mirror images of one another (the mirror images are in the same line color). **(C)** Waveform of lateral bending when *Dipsosaurus dorsalis* exhibits intermediate waves, adapted from Ritter (1992), with permission. The figure was made by a similar method to that applied for **(B)**. The numbered lines indicate points of minimal lateral displacement in each stick figure.

Some species of lizards, such as *Dipsosaurus dorsalis*, also show speed-dependent gait transitions (Ritter, 1992). They use standing waves at lower speeds and traveling waves at higher speeds, similar to the salamander's gait. Interestingly, they also use “intermediate” waves at intermediate speeds in between the speeds for standing and traveling waves. The waveform has attributes of both standing and traveling waves. To investigate whether the proposed model can reproduce these gait patterns, we performed a simulation by setting the parameter ω to 2.3π, that is, in between standing and traveling waves; and the remaining parameters were the same as those used in section 3.1. Figure 6 and Supplementary Video 2 present the results. Figure 6A shows the diagram for lateral bending and the gait diagram. The footfall pattern was a L-S walk; the mean and SD of the duty factor were 66.8 and 0.23%, respectively; the mean and SD of the diagonality were 29.0 and 0.35%, respectively. These values were calculated from the 10 trials. The body flexion duration moves posteriorly but not continuously. The wave propagation has an irregular point at the hip (*j* = 7). Figures 6B,C show the lateral displacement of each body part toward the moving direction of the simulated robot and the lizard, *D. dorsalis*, respectively. In Figure 6B, the minimal lateral displacement point moves posteriorly, similar to traveling waves. However, there are several points at the same position (posterior to the shoulder) as if the nodes were present, similar to standing waves. Therefore, the waveform has attributes of both standing and traveling waves; thus, intermediate waves emerge. Figure 6C shows the waveform when the lizard exhibits intermediate waves. The numbered lines indicate the minimal lateral displacement points. The points moved posteriorly, and some of them were within a restricted portion of the mid-trunk. The tendency is qualitatively similar to that of the simulation results. Therefore, the proposed model without any modified parameter except for the control parameter ω reproduced gait patterns exhibited by the *D. dorsalis*.

#### L-S Walk With Traveling Waves

**Figure 7:**
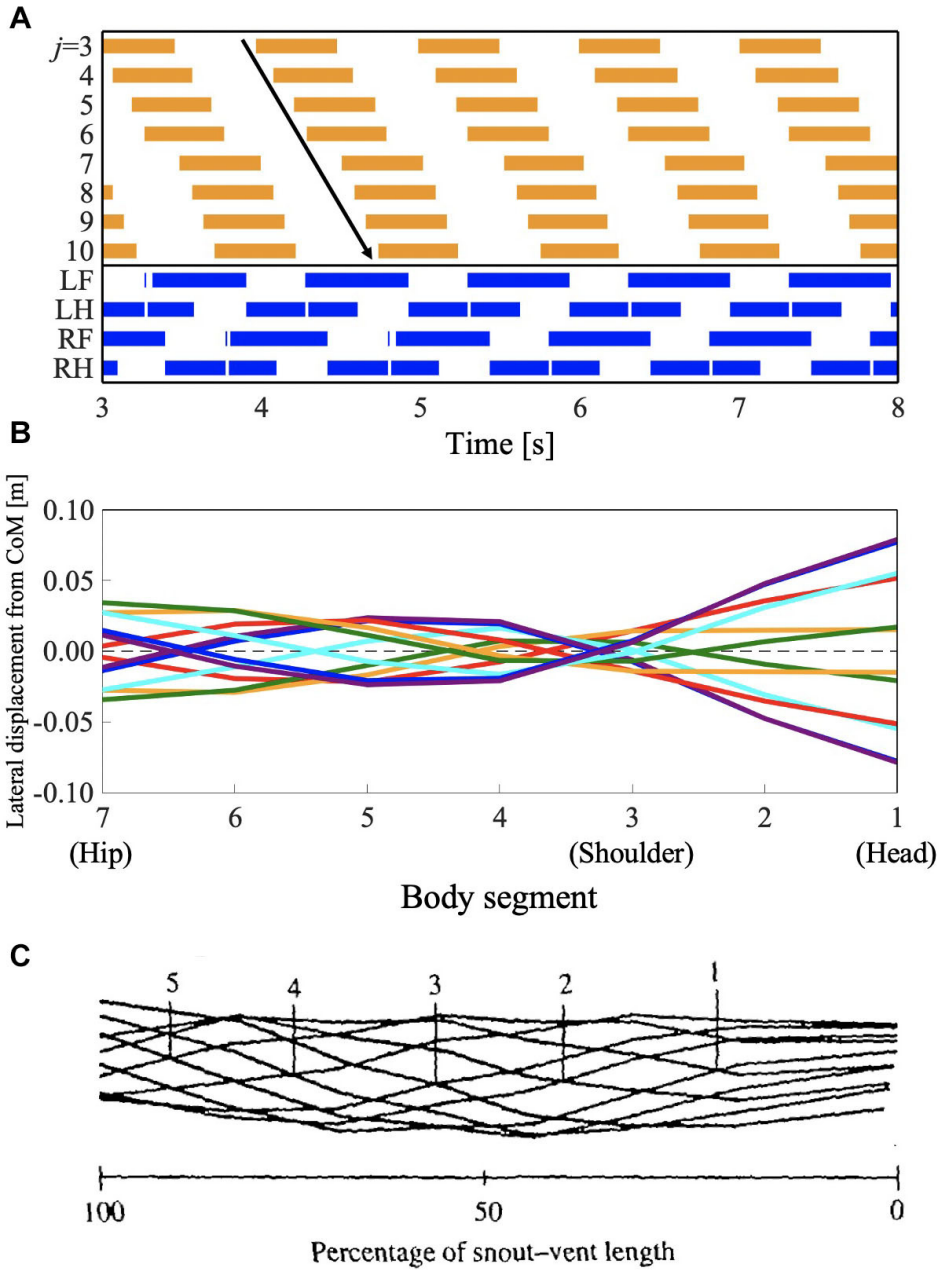
Lateral-sequence walking gait with traveling waves of lateral bending. The proposed model (with modified feedback gain parameters) reproduced the gait pattern, as listed in Table 1. **(A)** The upper graph represents the lateral flexion of the trunk joint, wherein the colored region denotes the period when the trunk joint bends to the right ($\theta_{j}^{b}>0$). The lower graph represents the gait diagram, wherein the colored region denotes the period when the foot is in contact with the ground (*N*_*i*_ > 0). **(B)** Waveform of lateral bending of the simulated robot. The superimposed figure was made from 12 stick figures. The stick figures were produced by connecting the lateral displacement of body segments from the center of mass (CoM). The moment of each stick figure is time-shifted every 1/12 gait cycle, and the line colors show the order of the stick figure (red: 1st and 7th; orange: 2nd and 8th; green: 3rd and 9th; cyan: 4th and 10th; blue: 5th and 11th; violet: 6th and 12th). Note that the 1st and 7th stick figures are time-shifted by a half period of the gait cycle, thereby being mirror images of one another (the mirror images are in the same line color). **(C)** Waveform of lateral bending when *Gerrhonotus kingii* exhibits traveling waves, adapted from Ritter (1992), with permission. The figure was made by a similar method to that applied for **(B)**. The numbered lines indicate points of minimal lateral displacement in each stick figure.

The salamander uses a traveling wave when performing a walking trot, but they have not been found to use traveling waves when using other slower walking gaits (Edwards, 1977). However, some lizards such as *G. kingii*, exhibit traveling waves, even at the lowest speed (Ritter, 1992). To investigate whether the proposed model can reproduce such gait patterns, we performed a simulation by setting the parameter ω to 2.3π, and the feedback gain from limb-to-body σ_*LB*_ and body-to-body σ_*BB*_ to lower values than those used in section 3.1 (σ_*LB*_ = 4.5 and σ_*BB*_ = 5.0, respectively). Figure 7 and Supplementary Video 3 show the results. Figure 7A shows the diagram for lateral bending and the gait diagram. The footfall pattern was a L-S walk; the mean and SD of the duty factor were 64.2% and 5.28 × 10^−2^, respectively. The mean and SD of the diagonality were 38.7 and 0.13%, respectively. These values were calculated from the 10 trials. The body flexion duration moves posteriorly and continuously. Figures 7B,C show the lateral displacement of each body part toward the moving direction of the simulated robot and the lizard, *G. kingii*, respectively. In Figure 6B, the minimal lateral displacement point moves posteriorly and continuously. The waveform is a traveling wave in which no node is present. Figure 6C shows the waveform when the lizard exhibits a traveling wave. The numbered lines indicate the minimal lateral displacement points. The figure shows that the points move posteriorly and continuously. The tendency is qualitatively similar to that of the simulation results. Therefore, the proposed model (with modified feedback gain parameters) reproduced the gait patterns exhibited by the *G. kingii*.

### 3.3. Effect of Sensory Feedback Strength on Gait Patterns

We performed simulations by changing various parameter sets, particularly, the feedback strengths, to specify the determinants of the gait patterns. For a quantitative gait evaluation, we used the two indices: diagonality and waveform index. The waveform index *W* was derived based on the gait evaluation method proposed by Kano et al. (2014) as follows:

(8) $$W=D_{std}-D_{trv},$$

(9) $$D_{x}=\underset{\Theta \in [0,2\pi ]}{\min}|r-e^{\text{i}\Theta }r_{x}|,$$

(10) $$r=\{e^{\text{i}\Phi_{3}},e^{\text{i}\Phi_{4}},e^{\text{i}\Phi_{5}},e^{\text{i}\Phi_{6}},e^{\text{i}\Phi_{7}},e^{\text{i}\Phi_{8}},e^{\text{i}\Phi_{9}},e^{\text{i}\Phi_{10}}\},$$

(11) $$r_{std}=\{0,0,0,0,e^{\text{i}\pi },e^{\text{i}\pi },e^{\text{i}\pi },e^{\text{i}\pi }\},$$

(12) $$r_{trv}=\{0,e^{-\text{i}\frac{\pi }{4}},e^{-\text{i}\frac{2\pi }{4}},e^{-\text{i}\frac{3\pi }{4}},e^{-\text{i}\frac{4\pi }{4}},e^{-\text{i}\frac{5\pi }{4}},e^{-\text{i}\frac{6\pi }{4}},e^{-\text{i}\frac{7\pi }{4}}\},$$

where *W* denotes the waveform index. *D*_*std*_ and *D*_*trv*_ are the intergait distances of standing and traveling waves, respectively. **r**, **r_std_**, and **r_trv_** are the phase relationships between the trunk-joint angles (*j* = 3−10) of the exhibited wave, the standing wave, and the traveling wave, respectively. The phase of the trunk joint Φ_*j*_ can be defined by the timing of lateral flexion of the trunk joint; for example, Φ_*j*_ = 0 is the timing when the trunk joint bends to the right from the neutral angle ($\theta_{j}^{b}=0$). Intergait distance is a measure for gait evaluation proposed by Kano et al. (2014). The distance shows the similarity of the specific gaits. For example, when a standing wave emerges, *D*_*std*_ is lower and *D*_*trv*_ is higher. Conversely, when a traveling wave emerges, *D*_*std*_ is higher and *D*_*trv*_ is lower. Therefore, when the waveform index *W* is positive, traveling waves emerge, when *W* is negative, standing waves emerge; and when *W* = 0, intermediate waves emerge. For further details of the derivation process, please refer to Kano et al. (2014).

**Figure 8:**
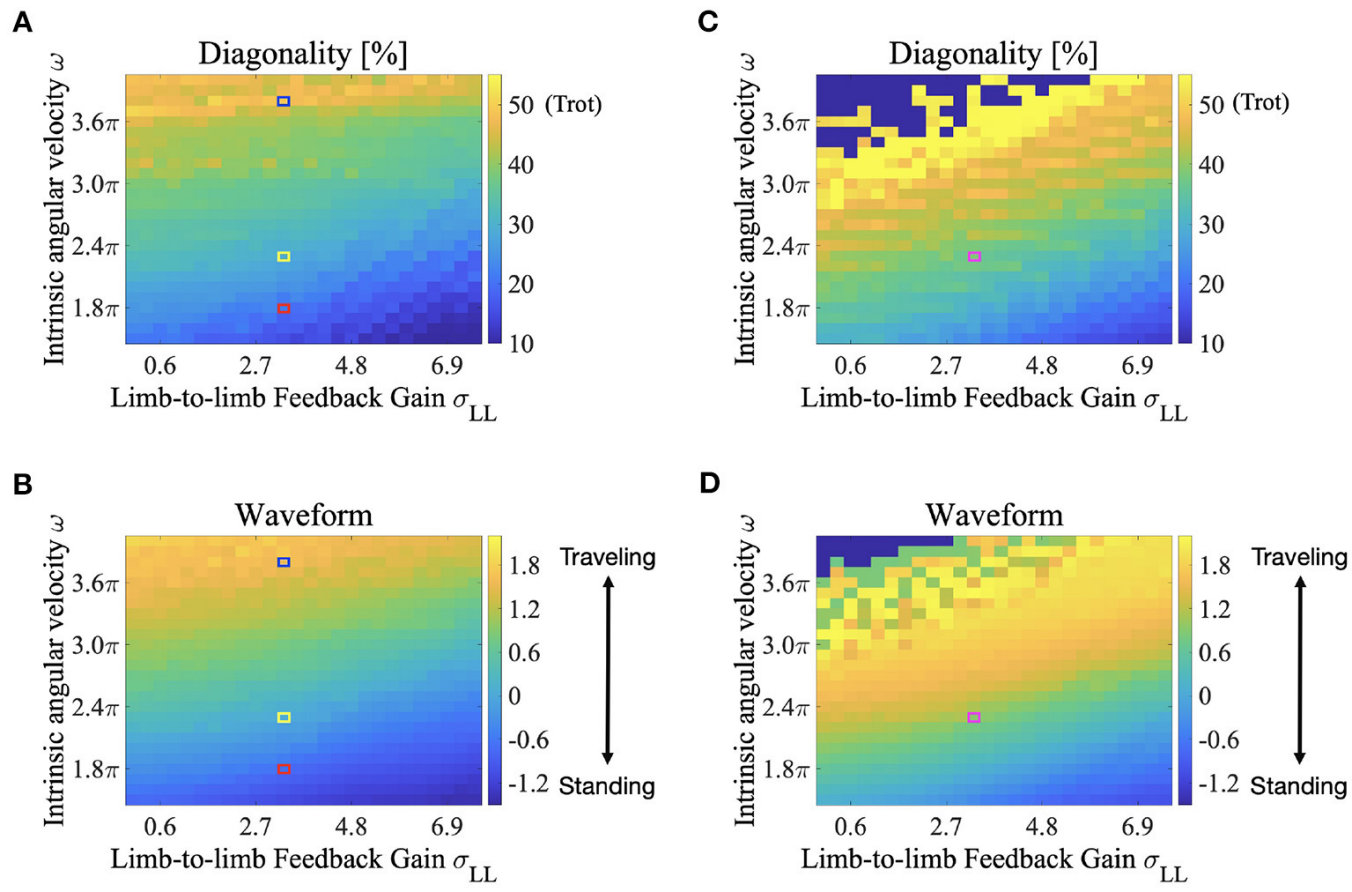
Color maps showing the two indices; diagonality and the waveform index, when the intrinsic angular velocity ω is between 1.5π and 4.0π, and the feedback gain from limb-to-limb σ_*LL*_ is between 0.00 and 7.50. **(A)** diagonality (σ_*LB*_ = 7.0, σ_*BB*_ = 7.7), **(B)** waveform index (σ_*LB*_ = 7.0, σ_*BB*_ = 7.7), **(C)** diagonality (σ_*LB*_ = 4.5, σ_*BB*_ = 5.0), **(D)** waveform index (σ_*LB*_ = 4.5, σ_*BB*_ = 5.0). In **(A,C)**, the brighter region indicates a higher diagonality. In **(B,D)**, the brighter region shows that a traveling wave emerges. The fluctuation in the upper left part of **(C,D)** indicates that an unstable locomotion emerged, and the gait was not evaluated correctly. The squared regions indicate the parameter sets used in the other experiments (red: Figure 4 for ω = 1.8π described in section 3.1; blue: Figure 4 for ω = 3.8π described in section 3.1; yellow: Figure 6 described in section 3.2; and purple: Figure 7 described in section 3.2).

The color maps in Figure 8 show the two indices when the intrinsic angular velocity ω is between 1.5π and 4.0π and the feedback gain from limb-to-limb σ_*LL*_ is between 0.00 and 7.50. In Figures 8B,D, the control parameters σ_*LB*_ and σ_*BB*_ are 7.0 and 7.7, respectively. In Figures 8C,D, the control parameters σ_*LB*_ and σ_*BB*_ are 4.5 and 5.0, respectively. The fluctuation in the upper left part of Figures 8C,D indicates that an unstable locomotion emerged, and the gait was not evaluated correctly. The squared regions indicate the parameter sets used in the other experiments (red: Figure 4 for ω = 1.8π described in section 3.1; blue: Figure 4 for ω = 3.8π described in section 3.1; yellow: Figure 6 described in section 3.2; and purple: Figure 7 described in section 3.2).

According to Figures 8A,B, both the diagonality and the index *W* increases as ω increases. Therefore, a walking trot with a traveling wave emerges for large values of ω. Conversely, both indices decrease as σ_*LL*_ increases, and a L-S walk with a standing wave emerges for large values of σ_*LL*_. Intermediate waves emerge for intermediate values of ω. Meanwhile, the values of both the diagonality and index *W* in Figures 8C,D are generally larger than those in Figures 8A,B. Therefore, for small σ_*LB*_ and σ_*BB*_, a walking trot with a traveling wave emerges even at a relatively small value of ω. This tendency is qualitatively consistent with the behavior of G. kingii using traveling waves exclusively, even at extremely slow speeds (Ritter, 1992).

### 3.4. Effect of Body Size and Mass on Gait Patterns

**Figure 9:**
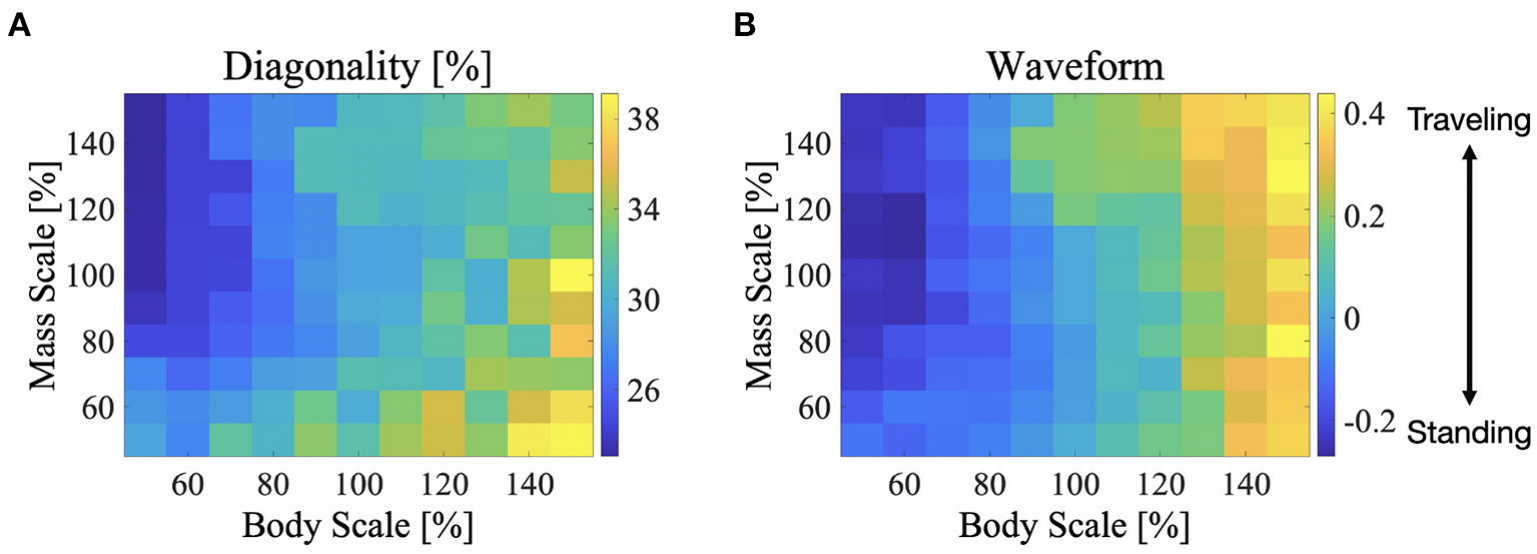
Color maps showing the two indices; diagonality and the waveform index, when the body size and mass are between 50 and 150%, **(A)** diagonality, **(B)** waveform index. In **(A)**, the brighter region indicates a higher diagonality. In **(B)**, the brighter region shows that the waveform is relatively similar to the traveling waves. The control parameter set was used in Figure 6 is described in section 3.2; for the L-S walk with intermediate waves.

We performed simulations by changing the body size and weight, and investigated the effect of these parameters on the exhibited gait patterns. This experiment used the same parameter set used in Figure 6 which is described in section 3.2 (i.e., for the L-S walk with intermediate waves). The color maps in Figure 9 show the two indices; diagonality and waveform index, when the body size and weight are between 50 and 150% of those, as listed in Table 1. Figure 9A shows that the diagonality tends to be higher for a larger body size and be lower for a heavier body. Figure 9B shows that the waveform index tends to be higher; that is, the waveform is relatively similar to traveling waves, for a larger body size.

**Table 1:** Parameter values of employed in the simulations.

Common parameters	Body length [m]		0.60
	Shoulder/hip length [m]		0.06
	Leg length [m]		0.06
	Body mass [kg]		2.85
	Joint mass [kg]	*m*_*J*_	0.10
	Link mass [kg]	*m*_*L*_	0.05
	Foot mass [kg]	*m*_*F*_	1.0 × 10^−5^
	Spring coefficient of trunk actuators [N/m]		1.5
	Damper coefficient of trunk actuators [(N·s)/m]		0.5
	Leg amplitude in yaw direction [rad]	$C_{0}^{y}$	π/9
	Leg amplitude in roll direction [rad]	$C_{0}^{r}$	π/9
	Gain for limb-to-limb feedback [rad/s]	σ_*LL*_	3.30
	Gain for limb-to-limb feedback [1/N]	ρ_*LL*_	0.10
	Gain for body-to-limb feedback [rad/s]	σ_*BL*_	4.00
	Gain for body-to-limb feedback [1/(N · m)]	ρ_*BL*_	0.20
	Gain for limb-to-body feedback [1/N]	ρ_*LB*_	0.20
	Gain for body-to-body feedback [1/rad]	ρ_*BB*_	1.00
Section 3.1	Intrinsic angular velocity [rad/s]	ω	1.8π → 3.8π at 16 s
			3.8π → 1.8π at 22 s
	Gain for limb-to-body feedback [N·m]	σ_*LB*_	7.00
	Gain for body-to-body feedback [N·m]	σ_*BB*_	7.70
Section 3.2			
Lateral-sequence walk	Intrinsic angular velocity [rad/s]	ω	2.3π
with intermediate wave	Gain for limb-to-body feedback [N·m]	σ_*LB*_	7.00
	Gain for body-to-body feedback [N·m]	σ_*BB*_	7.70
Lateral-sequence walk	Intrinsic angular velocity [rad/s]	ω	2.3π
with traveling wave	Gain for limb-to-body feedback [N·m]	σ_*LB*_	4.50
	Gain for body-to-body feedback [N·m]	σ_*BB*_	5.00

## 4. Discussion

To the best of our knowledge, this is the first study to demonstrate the spontaneous gait transition from lateral sequence walking with standing body waves, to walking trot with traveling body waves, in sprawling quadruped locomotion. The gait transition was achieved by changing only one parameter ω, which is related to a command from the brain. In the previous studies, Ijspeert et al. (2007) reproduced the salamander gait transitions from walking to swimming. This study also reproduced the standing- and traveling-wave patterns by modulating the strength of the descending command. However, the traveling waves were used for swimming but not for walking, and the walking pattern was uniquely determined by inter-oscillator couplings between the limbs and body CPGs. Harischandra et al. (2011) proposed a CPG model utilizing sensory feedback based on the Ijspeert's model and showed the gait transition from walk to trot. That study suggested that sensory modulation has an essential role for gait transition. However, because the body–limb coordination patterns were predetermined by inter-oscillator couplings, the transition of the bending patterns of the body trunk was not reproduced. In contrast, we designed a CPG controller based on sensory couplings through bidirectional feedback between the limbs and body without inter-oscillator couplings, and we demonstrated that the controller can reproduce flexible body–limb coordination patterns. This result suggests that the proposed sensory feedback mechanisms could play an important role in sprawling quadruped locomotion.

The proposed model changes the footfall pattern in response to the control and body parameters. Specifically, diagonality tends to be higher when the leg phase oscillator has a higher frequency. For example, the higher the intrinsic angular velocity of the oscillator ω, the higher the diagonality (Figures 8A,C). Meanwhile, the higher feedback gain of limb-to-limb σ_*LL*_ and body-to-limb σ_*BL*_ tend to have lower diagonality owing to the effect of the phase modification that decreases the phase frequency. Similarly, a heavier body tends to have a lower diagonality because a heavier body can obtain a higher reaction force *N*_*i*_ that enhances the limb-to-limb feedback. Furthermore, the higher feedback gain of limb-to-body σ_*LB*_ and body-to-body σ_*BB*_ tend to have lower diagonality because the feedback gain is related to the generated torque at the trunk, which enhances the body-to-limb feedback. Owing to the close interactions between the sensory feedback mechanisms, the proposed model generates flexible footfall patterns.

Sprawling quadruped animals use various body bending patterns. However, the mechanisms responsible for generating flexible bending patterns remain unclear. In this article, we presented a potential solution to generate various bending patterns. Our proposed model coordinates axial movements using curvature derivative control and sensory feedback from the legs. Curvature derivative control causes the angle of the trunk joint to follow that of the anterior trunk joint. Therefore, the control shapes a traveling wave of axial movements. Because the control gain (here is σ_*BB*_) is related to the follow-up speed, the higher the feedback gain, the faster the wave speed of the traveling waves. Meanwhile, the feedback from the legs imposes bending of the trunk joint at the shoulder (*j* = 3) and hip (*j* = 7) in response to the ground contacts. Given that the footfall timings of diagonally opposite feet are roughly synchronized, the feedback tends to cause the bending of the shoulder antiphase with respect to that of the hip. As a result, a standing wave with nodes at the shoulder and hip emerges for a large feedback gain of curvature derivative control σ_*BB*_, that is, when the wave speed is higher. When the σ_*BB*_ is lower, the wave speed is lower, and a traveling wave emerges. The intermediate wave emerges in the condition between those of the standing wave and traveling waves. Based on these mechanisms, the proposed model generates flexible bending patterns. Furthermore, increasing the body size has a similar effect of reducing the body-to-body feedback gain (Figure 9B) because a larger body size has a higher inertia that delays the wave speed of the body bending.

We hypothesized that a salamander possesses load and stretch sensors at each body part and that sensory information is transmitted to nearby body parts. At present, there is no definitive neurophysiological evidence for the proposed sensory feedback mechanism. However, several biological findings suggest that the proposed mechanism possibly exists. First, it has been reported that the salamander's body and limbs have mechanoreceptors (Chevallier et al., 2008; Ryczko et al., 2020). Second, similar feedback mechanisms were reported for other vertebrates. Specifically, cats utilize signals related to the force in leg muscles to initiate the transition from the stance to swing phase in each leg (Pearson et al., 2006), while lampreys utilize stretch receptors along the trunk to coordinate axial movements (Grillner, 1996). Third, the neural circuits for limb movements are located in particular vertebrae above and below the axial trunk network (Bicanski et al., 2013b). Therefore, sharing sensory signals among nearby body parts is feasible. Further biological studies are required to prove the validity of the proposed mechanisms. In addition, direct inter-oscillator couplings are known to exist within the salamander spinal cord, in particular in the axial networks (Ryczko et al., 2010), whereas in this study we purposely removed them in order to focus on sensory-driven synchronization mechanisms. Future studies should investigate this further once the actual neural circuits of the salamander spinal cord are better known. The finding of this study suggests that the role of inter-oscillator coupling in shaping the locomotor patterns might be less important than previously thought, compared to sensory-driven mechanisms.

In the future, we aim to develop a salamander robot and verify the proposed model in the real world. We will investigate the locomotion speed and cost efficiency for various gait patterns and contribution of the proposed sensory feedback mechanisms. This will contribute to an understanding of the merits of gait transitions in sprawling locomotion. Furthermore, we will investigate the robustness of ground property changes. Although this study used flat terrain as the experimental environment, we expect that the proposed sensory feedback mechanisms have some adaptability toward various ground properties, such as a granular surface and gravel road, owing to the body–limb sensory feedback mechanisms. Finally, we would like to elucidate a common principle underlying body–limb coordination by studying other animals. We have already proposed models for body–limb coordination of sea roaches (Kano et al., 2019) and quadrupeds that exhibit cheetah-like galloping (Fukuhara et al., 2020). Based on these studies, we aim to find commonalities to various legged animals, and establish a universal control framework for legged robots with high robustness and adaptability.

## Data Availability Statement

The original contributions presented in the study are included in the article/Supplementary Material, further inquiries can be directed to the corresponding author/s.

## Author Contributions

SS, AIj, and AIs designed the study. AIs supervised the project. SS, TK, and AIs designed the mathematical model. SS carried out the simulation experiments and the initial draft of the manuscript. All authors are involved in revising the manuscript.

## Conflict of Interest

The authors declare that the research was conducted in the absence of any commercial or financial relationships that could be construed as a potential conflict of interest.

## Publisher's Note

All claims expressed in this article are solely those of the authors and do not necessarily represent those of their affiliated organizations, or those of the publisher, the editors and the reviewers. Any product that may be evaluated in this article, or claim that may be made by its manufacturer, is not guaranteed or endorsed by the publisher.
